# Program Report: The Advanced Technology Program: A New Role for NIST in Accelerating the Development of Commercially Important Technologies

**DOI:** 10.6028/jres.096.036

**Published:** 1991

**Authors:** Brian C. Belanger, George A. Uriano, Raymond G. Kammer

**Affiliations:** National Institute of Standards and Technology, Gaithersburg, MD 20899

**Keywords:** advanced technology, cooperative agreements, extra mural, generic technology, industrial competitiveness

## Abstract

The Advanced Technology Program (ATP) is a new extra mural program operated by the National Institute of Standards and Technology (NIST) for the Department of Commerce’s Technology Administration. The ATP will help enhance U.S. competitiveness by funding the development of pre-competitive, generic technologies in partnership with industry. This paper describes the ATP, the first ATP awards made by NIST, and the prognosis for the success of the ATP.

## 1. Introduction

The National Institute of Standards and Technology (NIST), formerly the National Bureau of Standards (NBS), has a well-deserved reputation for working effectively with industry. However, until recently, NIST assistance to industry involved primarily measurement and standards expertise developed within NIST laboratories and disseminated via mechanisms such as calibration services, standard reference materials, standard reference data, and information needed for the establishment of technical specifications. As a result of the Omnibus Trade and Competitiveness Act of 1988, NIST now has an augmented role in enhancing industrial competitiveness and promoting U.S. economic growth. The Advanced Technology Program is one of several new responsibilities assigned to NIST that contribute to this increased emphasis on industrial competitiveness. (Others include the Malcolm Baldrige National Quality Award, the Manufacturing Technology Centers Program, and the State Technology Extension Program.)

The ATP provides research and development grants in the form of cooperative agreements to individual companies, independent research institutes, or joint ventures. Awards are for the development of precompetitive, generic technology. These terms are defined as follows:

*Generic Technology:* A concept, component, or process, or the further investigation of scientific phenomena, that has the potential to be applied to a broad range of products or processes. A generic technology may require subsequent research and development for commercial application.

*Precompetitive Technology:* R&D activities up to the stage where technical uncertainties are sufficiently reduced to permit assessment of commercial potential and prior to development of application-specific commercial prototypes. At this stage, for example, results can be shared within a consortium that can include potential competitors without reducing the incentives for individual firms to develop and market commercial products and processes based on the results.

During the Congressional deliberations on the Omnibus Trade and Competitiveness Act of 1988, there was considerable debate regarding the proper role for the Federal government in supporting civilian technology R&D. Today there is a consensus within both the Administration and the Congress that support for precompetitive, generic technology development is an appropriate role for the Federal government. For example, the September 26, 1990 report, “U.S. Technology Policy,” prepared by the President’s Office of Science and Technology Policy, stated (p. 5) that it is the responsibility of the Federal government to “participate with the private sector in precompetitive research on generic, enabling technologies that have the potential to contribute to a broad range of government and commercial applications.”

In a March 7, 1990 speech to the American Electronics Association, President Bush said, “This Administration is also committed to working with you in the critical precompetitive development stage where the basic discoveries are converted into generic technologies that support both our economic competitiveness and our national security. Here again we can help to level the international playing field on which you compete.” At the November 13, 1990 ceremony to present the National Medals of Science and of Technology, the President said, “Today our government must help carry that research forward and contribute to the development of generic technologies that build on basic discoveries. If America is to maintain and strengthen our competitive position, we must continue not only to create new technologies but learn to more effectively translate those technologies into commercial products. In this way, we can help leverage the R&D of the private sector, helping whole industries advance in an increasingly competitive global market.”

In keeping with these guidelines, projects supported by the ATP involve R&D at a stage between basic research and specific commercial product development. To be appropriate for ATP funding, a project must be characterized by challenging technical problems that must be solved before further steps towards commercialization can take place. While the ATP supports projects having high technical risk, the potential benefits to the U.S. economy must be commensurate with that high risk.

## 2. ATP Eligibility

Two categories of applicants may apply for ATP awards: single applicants (U.S. companies or independent research institutes), and joint ventures. The rules for participation differ for the two types of applicants as noted below. (Most features of the ATP rule reflect explicit provisions of the ATP legislation.)

### 2.1 Single Applicants

Single applicants may be either companies or “independent research institutes.” (Examples of well-known independent research institutes are the Stanford Research Institute (SRI), the Battelle Memorial Institute, and the Southwest Research Institute.) Universities and government laboratories may not apply directly to the ATP, although they may participate as subcontractors to single applicants or in joint ventures — assuming the minimum joint venture eligibility requirements have been met, as described in the next section.

Awards to single applicants cannot exceed $2 million over a 3-year period. NIST cannot pay indirect costs for single applicants, therefore they must absorb overhead costs themselves or find other sponsors willing to cover these costs.

### 2.2 Joint Ventures

The ATP may fund joint ventures for up to 5 years. The dollar amount of any joint venture award is limited only by the total funding available for the ATP in any given year. Joint ventures must provide matching funds of at least 50% of the total cost of the project.^1^ Federal funds, whatever the source, do not count toward the matching funds requirement for joint ventures. Accordingly, the matching funds must be provided by the participants, by state and local governments, by private investors, or by some combination of these sources.

To be eligible for ATP awards, joint R&D ventures must consist of at least two organizations that agree to work together on the proposed R&D program. Both must be eligible to apply alone, and both must contribute toward the matching funds. Thus, a minimum-size joint venture must have at least two companies, two independent research institutes, or one of each, both of which are doing R&D and contributing toward the match. Joint ventures may also include any number of additional companies or independent research institutes, universities, and/or government laboratories, each of which may or may not contribute to the matching funds requirement and/or participate in the research.

### 2.3 NIST Participation in ATP-Funded Projects

NIST encourages its scientists and engineers to collaborate with joint venture or single applicant projects funded by the ATP, but NIST’s intramural laboratory programs cannot receive funds from an ATP-funded project.^2^ Although encouraged, collaboration with NIST is not a selection criterion, so a proposal featuring such collaboration will not score higher than one that does not.

### 2.4 Participation in the ATP by Foreign-Owned Companies

Congress has debated whether foreign-owned firms should be allowed to compete for ATP awards, and if so, under what conditions. No restrictions regarding the nationality of corporate ownership were included in the legislation that established the ATP other than statements to the effect that the program was intended to help “U.S. businesses.” The legislation did not define “U.S. business.” During the first competition, NIST interpreted this provision of the law such that companies committed to conducting the proposed R&D in the United States and providing evidence that they would also carry out in the U.S. subsequent manufacturing resulting from the R&D, were eligible—whatever the corporate owners’ nationality.

The Fiscal Year 1991 Department of Commerce Appropriations Bill contained language that placed additional restrictions on foreign-owned companies that wished to participate in the ATP. Since an appropriation bill is only valid for the year of the appropriation, this legislative language will not apply to future ATP appropriations unless the Congress includes it in future authorization and/or appropriations bills (which seems likely). The language says that the ATP can fund only companies that make investments in the United States in research, development, and manufacturing; make significant contributions to employment in the United States; and agree to promote manufacturing within the United States of products resulting from the ATP-funded project.

Such companies must either 1) be a U.S. company (meaning more than 50%-owned by U.S. citizens), or 2) the Secretary of Commerce must determine that the applicant has a parent company incorporated in a country that affords U.S. companies equal access to programs analogous to the ATP, and affords adequate and effective protection for the intellectual property rights of U.S.-owned companies.

## 3. Proposal Evaluation

### 3.1 Selection Criteria

ATP proposals are judged on five selection criteria:

- Scientific and technical merit (20%)
  – Quality and innovativeness of the proposal
  – Appropriateness of the technical risk and feasibility of the project (This factor addresses whether the degree of risk is commensurate with the potential payoff. Note that the ATP is not adverse to high risk where the payoff is correspondingly high.)
  – Coherency of technical plan
  – Systems integration and multi-disciplinary planning (This factor involves the appropriate use of “concurrent engineering.”)
- Broad-based (commercial) benefits (20%)
  – Potential broad impact on U.S. technology and knowledge base
  – Potential to improve U.S. economic growth and productivity of a broad spectrum of sectors or businesses
  – Timeliness of the proposal
- Technology Transfer Benefits of the Proposal (20%)
  – Evidence that if the project is successful, the participants will pursue further development of the technology toward commercial application
  – Addresses technology transfer requirements to assure prompt and widespread use
- Experience and Qualifications of Proposing Organization (20%)
  – Staffing, facilities, equipment, other resources
  – Quality and appropriateness of full-time technical staff
  – Design and manufacturing tools adequate for laboratory prototype development
- Proposer’s Level of Commitment and Organizational Structure (20%)
  – Level of commitment (contribution of personnel, equipment, facilities, etc.)
  – For joint ventures, appropriateness of structure (i.e., horizontal vs. vertical integration)
  – For joint ventures, appropriate participation by small businesses.
  – Evidence of commitment to complete program and continue beyond period of Federal funding
  – Potential return to the Government (royalty and licensing fees)

### 3.2 The Selection Process

The “Selecting Official” for the first competition was Dr. John W. Lyons, NIST’s Director. He appointed a 10-person Source Evaluation Board (SEB). The SEB was responsible for ranking the proposals against the selection criteria and recommending those most deserving of funding.

The SEB included senior level NIST technical managers whose backgrounds reflected the disciplines represented by the proposals, (e.g., chemistry, physics, electrical engineering, computer science, materials science, etc.), as well as managers with considerable experience in business planning, business development, finance, and intellectual property. The SEB used technical experts and business experts to prepare written reviews of proposals. All reviewers were screened carefully to eliminate conflicts of interest and were required to sign non-disclosure agreements.

The SEB considered carefully the recommendations made by the technical and business reviewers, but was not bound by them. In ranking proposals, the SEB discussed each reviewer’s comments on the merits (or lack thereof) of each proposal, but exercised its independent judgment as well.

In the first competition, 249 proposals were received. As a first step, the SEB screened them to ensure compliance with the solicitation requirements. For example, proposals submitted directly by universities were rejected because the ATP legislation does not allow direct funding of universities. Similarly, proposals submitted by joint ventures not providing more than 50% matching funds were rejected, as were proposals from single applicants that did not agree to pay indirect costs. Forty proposals were rejected during the initial screening stage.

The remaining proposals were subjected to a thorough technical review by 397 different technical experts from NIST, other government agencies and laboratories, universities, and the private sector. Federal agencies and laboratories that helped with the reviews included the Defense Advanced Research Projects Agency (DARPA), the Naval Research Laboratory, the Goddard Space Flight Center, the Department of Energy (DOE) and the DOE national laboratories, the Air Force Office of Scientific Research, the Army Corps of Engineers, the Office of Naval Research, and the National Institutes of Health.

The proposals with the highest technical merit (approximately the top third) were submitted to the business reviewers. Most business reviewers, while drawn from different backgrounds than the technical reviewers, also had strong technical qualifications. They included: a retired dean of a business school, high-tech venture capitalists, vice presidents of engineering or R&D for large, high-tech corporations, and presidents of smaller technology-intensive companies. These individuals generally had considerable experience in transferring technologies from the R&D laboratory to production.

Twenty-one proposals that scored highest in both the technical and business reviews were designated as semifinalist proposals. Each semi-finalist proposer presented an oral defense at NIST. During the oral defense, the SEB asked questions raised by the technical and business reviewers and requested clarifications of points not completely addressed in the written proposals. These oral defenses proved very helpful to the SEB in preparing the final rankings, because information surfaced that strengthened the case for funding certain proposals and weakened the case for others. While the SEB reserved the right to make site visits where appropriate, none were needed for any of the semifinalists.

## 4. Intellectual Property

Awardees are generally granted full title to intellectual property developed during ATP-funded projects. The Federal government retains a royalty-free, non-exclusive license to use the technology for government purposes, as is generally the case with Federally-funded programs. Current Federal laws and policies^3^ give the Federal government “march-in rights” that allows NIST to intercede if, after a reasonable period, the ATP awardee has failed to use the technology developed under the ATP project. There are also some restrictions under this act that limit the ability of the company or companies holding title to intellectual property developed during an ATP project to license it overseas without the permission of the Federal government.

## 5. Awards

The ATP announced its first awards on March 5, 1991. Appendix A provides a listing of the 11 projects selected in the first competition. They included five joint ventures and six single applicants. Small businesses participate in all of the joint ventures (and lead two of them), and four of the six single applicant awardees are small businesses. In the press release announcing the first awards, Commerce Secretary Robert A. Mosbacher said the awards “could lead to the birth of revolutionary products and processes in key U.S. industries and help boost the country’s trade and competitiveness.”

Several new joint R&D ventures formed specifically in response to the ATP solicitation. Because of the matching fund requirement (for joint ventures) and the indirect cost contribution (for single applicants), the approximately $9 million provided by NIST for the first year of the projects selected is highly leveraged. A total of $45.8 million was requested from the ATP over the duration of the 11 projects selected. The applicants’ cost-sharing amounted to an additional $51.7 million. Thus R&D valued at nearly $100 million will result from these awards over a 5-year period.

## 6. The Future

Congress appropriated $35.9 million for the ATP in Fiscal Year 1991 and the President’s budget for FY 1992 also requests $35.9 million. Initial reactions to the ATP’s first competition by the Congress and U.S. industry appear favorable, and thus the program appears to have a bright future. The next request for proposals will be issued in the summer of 1991. Some of the $35.9 million in FY 1991 funds will be used to fund the second year of the ongoing projects, but there should be approximately $20 million available for beginning new projects.

By the mid-1990s, an assessment will be available of the degree of success of the first ATP-funded projects. Certainly, not all ATP projects will be successful. Since the ATP intentionally funds high-risk, potentially high-leverage projects, a number will undoubtedly fail. However, NIST is optimistic that enough will succeed to justify the expenditures. NIST is currently developing measures of success for the ATP. As experience is gained, NIST should become increasingly proficient at identifying and selecting high leverage projects that will contribute to enhanced competitiveness of U.S. businesses and promote U.S. economic growth.

For more information on the Advanced Technology Program, contact:

Advanced Technology Program,
A402 Administration Bldg.,
National Institute of Standards and Technology,
Gaithersburg, MD 20899
(301) 975-5187

## Footnotes

1 The term “matching funds” includes the following: 1) dollar contributions from state, county, city, company, or other sources; 2) the applicant’s share of revenue from licensing and royalties; 3) fees for services performed; 4) in-kind contributions of full-time personnel; 5) contributions of a pro rata share of part-time personnel that the Program deems essential to carrying out the proposed experimental work program and who devote at least 50% of their time to the program; and 6) in-kind value of equipment that the Program deems essential to carrying out the proposed experimental work program, which may include either the purchase cost of new equipment or the depreciated value of previously purchased equipment. (Refer to the ATP Rule, 15 CFR 295.2(e), for additional restrictions on matching funds.)

2 Legislation has been considered by the Congress that would permit the NIST Director to utilize a small portion of the ATP budget to support work inside NIST to foster collaboration with projects selected for funding, where such work would enhance the chances for success of the ATP project and complement NIST’s internal R&D programs.

3 Awards under the program will follow the policies and procedures on ownership to inventions made under grants and cooperative agreements that are set out in the Bayh-Doyle Act—Public Law 96-517 (35 U.S.C. Chapter 18), the Presidential Memorandum on Government Patent Policy to the Heads of Executive Departments and Agencies Dated February 18, 1983, and Part 401 of Title 37 of the Code of Federal Regulations, as appropriate.

## 8. Appendix A. — The Eleven Proposals Selected for Awards in Competition 90-01

### Volume Holographic Mass Storage Subsystem

Microelectronics & Computer Technology Corporation (MCC) Radically new form of dense, ultra-fast computer memory storage (potentially replacing disk drives and magnetic tape) would have applications from laptop computers to supercomputers. Data stored as holographic images in photorefractive crystals. First-year request: $823 K

### Nonvolatile Magnetoresistive Semiconductor Technology

Nonvolatile Electronics, Inc. Magnetoresistive memory (MRAM) for computers is nonvolatile — the information is not lost when the power is shut off. Has applications across entire spectrum of microelectronic design. First-year request: $599 K

### Short-Wavelength Sources for Optical Recording

National Storage Industry Consortium Program to develop unique integrated, solid-state laser source for read/write heads of optical memory devices (like CDs). Identified in the Technology Administration’s Emerging Technologies report as key technical barrier. $50,000 initial grant contingent on further development of the joint venture, including Applied Magnetics, Bernoulli Optical Systems, Eastman Kodak, IBM, Maxoptix Corp., and the University of Arizona. Initial $50 K Planning Grant Awarded

### Tunable Deep UV and VUV Solid-State Laser Source

Light Age, Inc. Application of recent developments in laser technology to produce high-average-power ultraviolet lasers for medical, electronic materials processing, and scientific applications that are cheaper, safer, more reliable, and easier to use than current products. Light Age is an EPIC 100 firm, selected by the Commerce Department’s International Trade Administration for special export promotion. First-year request: $627 K

### New User-Interface for Computers Based on On-Line Recognition of Natural Handwriting

Communication Intelligence Corporation (CIC) Cursive handwriting input for computers “could well be as revolutionary as the original PC” (Fortune, 11 Feb 91). CIC plans a robust, natural handwriting-recognition system that is user-independent, a key limitation of most current systems. First-year request: $671 K

### Printed Wiring Board Interconnect Systems

National Center for Manufacturing Sciences, Inc. (NCMS) Used in virtually all electronics equipment. AT&T, Texas Instruments, Digital Equipment Corporation, and Hamilton Standard Interconnect are joined through NCMS to develop new PWB materials and production technology for a $25 billion world market. First-year request: $2,370 K

### Advanced Manufacturing Technology for Low-Cost Flat Panel Displays

Advanced Display Manufacturers of America Research Consortium A joint venture by several relatively small U.S. producers of flat-panel displays for computers —competing against large, vertically integrated Japanese firms—to develop key production and testing technologies for a multi-billion-dollar industry. First-year request: $1,251 K

### Fabrication and Testing of Precision Optics for Soft X-Ray Projection Lithography

AT&T Bell Laboratories Attacking a key problem limiting projection x-ray lithography (needed for future generations of very compact integrated circuits): the manufacture, testing, and assembly of relatively large-scale x-ray optics. An estimated 75 percent of the ATP funds will go to small-business subcontractors. First-year request: $955 K

### Solid-State Laser Technology for Point-Source X-Ray Lithography

Hampshire Instruments, Inc. & McDonnell Douglas Electronic Systems Co. Joint venture led by a small business to exploit recent developments in laser technology for a low-cost, high-performance x-ray source suitable for microlithography and the design and production of advanced integrated circuits. First-year request: $1,090 K

### Advanced Compensation Techniques for Enhancing Machine-Tool Accuracy

Saginaw Machine Systems, Inc. Seeks more general, economical solution to problem of correcting for errors in machining caused by thermal expansion and contraction of the machine tool. Wide potential applications in U.S. machine tool industry. Cost and accuracy are the two key selling points in machine tools. First-year request: $266 K

### Advanced Thallium Superconductor Technology

E. I. du Pont de Nemours & Co. Development of thin-film fabrication techniques for a new, proprietary high-temperature superconductor invented by Du Pont. Project includes developing fabrication techniques and creating representative superconducting electronic devices to demonstrate feasibility. First-year request: $370 K

